# Doctors during the COVID-19 pandemic: what are their duties and what is owed to them?

**DOI:** 10.1136/medethics-2020-106266

**Published:** 2020-10-15

**Authors:** Stephanie B Johnson, Frances Butcher

**Affiliations:** 1 Nuffield Department of Population Health, Department of Medicine, Univerity of Oxford Nuffield, Oxford, Oxfordshire, UK; 2 Honorary Specialty Registrar in Public Health, Oxford University Hospitals NHS Foundation Trust, Oxford, Oxfordshire, UK

**Keywords:** health personnel, clinical ethics, ethics, public health ethics

## Abstract

Doctors form an essential part of an effective response to the COVID-19 pandemic. We argue they have a duty to participate in pandemic response due to their special skills, but these skills vary between different doctors, and their duties are constrained by other competing rights. We conclude that while doctors should be encouraged to meet the demand for medical aid in the pandemic, those who make the sacrifices and increased efforts are owed reciprocal obligations in return. When reciprocal obligations are not met, doctors are further justified in opting out of specific tasks, as long as this is proportionate to the unmet obligation.

Doctors form an essential part of an effective response to the COVID-19 pandemic. They have critical roles in diagnosis, containment and treatment, and their commitment to treat despite increased personal risks is essential for a successful public health response.^1^ Frontline workers have been experiencing high work volume, personal risk and societal pressure to meet extraordinary demands for healthcare. Despite this traditional public health ethics has paid little attention to the protection of the rights of doctors.^2^

We will consider the role of doctors during the COVID-19 pandemic, focusing primarily on the British National Health Service (NHS), by answering the following four questions: what is the nature and scope of the duties of healthcare providers? To whom do these duties apply? What reciprocal obligations to doctors exist from their employers and patients? And what should doctors do when these reciprocal obligations are not met?

Although these questions are equally important to all healthcare professionals, we focus on doctors because it is important to distinguish that different healthcare professionals have different roles, and this may affect the extent of their occupational risks and duties. Further research on the role of nurses, physiotherapists and other health professionals should be undertaken but is beyond the scope of this article.

## Do doctors have a duty to treat in disease outbreaks and pandemics such as COVID-19?

With respect to moral theory, numerous grounds have been offered for the view that doctors have a duty to treat or an obligation to provide care to patients.^3^ With regards to pandemics, claims about the duties of doctors are most often grounded in so-called ‘special duties’ or ‘role related’ duties. In other words, by virtue of their profession, doctors have more stringent obligations of beneficence than most, and they have obligations to a specified group of persons (their patients) that non-medical personnel have no obligation to help.^4^ Clark^5^ argues that the duty can be justified with reference to: (A) special skills possessed by healthcare professionals, which mean that they are uniquely placed to provide aid, thereby increasing their obligation; (B) the individual’s freely made decision to enter the profession with the knowledge of what the job entails and the nature of the associated risks; and (C) the social contract between healthcare professionals and the society in which they work. However, it seems clear that the duty to treat cannot be ‘absolute’—that doctors have a duty to work regardless of the circumstance. Doctors have rights to protection and to care during an infectious disease outbreak, as do other members of society.^2^

In previous epidemics, arguments that have rationalised the abandonment of patients include futility when medicine is powerless to help and the depletion of finite human resources (healthcare workers) when physicians fall ill.^2 6^ Sokol^4^ points out that in times of crisis, the duties deriving from doctors’ multiple roles may often come into conflict, and the problem with many accounts of the duties of doctors is that they fail to acknowledge these tensions and to consider workers as multiple agents belonging to a broader community. Doctors, for instance, may have a duty to care for patients as well as a duty to care for their own families by protecting them (and hence themselves) from infection.^4^ Failure to account for the effects of interventions such as school closures on the healthcare workforce only exacerbate the problem of strained healthcare capacity by removing much needed members from the workforce.

### Special circumstances

Emerging threats of infectious diseases such as COVID-19 demand much more than that doctors continue to work as normal. Pandemics may necessitate longer hours (and corresponding increased exposure to the virus), potential quarantines and assignments outside one’s normal specialty.^3^ What distinguishes normal duty from acting beyond the call of duty is not always clear-cut.^7^ However, experience so far suggests that in the current epidemic doctors are subject to risk of illness,^8^ risk of death,^8^ fatigue from extended hours,^9^ moral distress (when being party to difficult treatment decisions, such as prioritisation of patients for ventilators)^9^ and potential legal and professional risks when be asked to work at the limits of their competencies.^10^

The 2003 SARS epidemic provided some important insights into the experience and pressures on healthcare workers during an epidemic, as well as highlighting some important gaps in ethical thinking and practice. Many of those who treated patients with SARS raised concerns about the protections that were provided to safeguard their own health and that of their family members.^11 12^ Some refused to attend SARS wards resulting in permanent dismissal, and some chose to leave the profession post-pandemic.^11 13^ Notably, it was recognised during SARS that there is no consensus as to how explicitly and stringently the requirements for the duty to care should be stated.^13 14^ Scholars recommended advance planning with local and national professional medical associations to obtain agreement about the extent of professional obligations in a pandemic.^11^ This was suggested to include the development of clear and unambiguous guidelines regarding the professional rights and responsibilities and the ethical duties and obligations of healthcare professionals during such outbreaks.^13^ Almost two decades later, there remains little consensus and clarity over reasonable expectations on the medical workforce. This is a grave failing.

## Is opting out justifiable?

If limits of the duty of care are not absolute but, rather, constrained by several factors defined by the strengths of competing rights and duties,^4^ it may be concluded that some doctors may be morally justified in opting out of frontline work. Opting out could be more easily justified if this frontline work extends beyond their area of expertise and/or places significant personal or physical burdens on them. For instance, an older doctor with diabetes may object to moving to frontline COVID-19 work, given the suggestion that higher mortality is associated with COVID-19 infection in those who are older or have comorbidities.^15^

There are two main objections to an ‘opt out’ policy. First, considerations of fairness. For each doctor who opts out, this places an additional burden on their colleagues. In particular, it could mean that burdens of the outbreak are placed on specific groups, such as young, childless doctors who will be overburdened and are likely to have less expertise. As Reid^16^ has pointed out, the health risk refused by one individual is left to be absorbed by someone else, either within the healthcare team or by society at large. Second, opting out may have a significant impact on patient trust, which has recognised importance in the efficacy of pandemic response.^17^ Others have argued that the need for health officials to be viewed as the experts, whose intentions and actions are in the best interest of the public, is critical to fostering trust.^18^ The medical profession is often described as having an implicit contract with society to provide medical help in times of crisis,^19^ which includes a reasonable and legitimate expectation by the public that doctors will respond in an infectious disease emergency.^13^ Trust in medical professionals, and the healthcare system as a whole, may be undermined were there a public perception that doctors were unwilling to act in the best interests of patients by failing to meet the extraordinary demand for healthcare.

While these are undesirable consequences that should be addressed, these objections are not strong enough moral justifications to pressure all doctors into working in circumstances beyond their expected role that they consider to be morally, psychologically or physically unacceptable.^4^ The moral, psychological and physical acceptability of frontline COVID-19 work is likely to be determined by a number of important factors, such as the level of personal risk of serious illness, personal circumstances, specialty, career stage and met/unmet reciprocal obligations (discussed further below).

## To whom do these duties apply?

While we have so far looked at the duty of care of doctors, this is not a homogenous group. All doctors have a duty (within limitations) to care for their patients, but an acutely unwell and infectious patient might not be within the normal range of practice of some specialties. If we compare an infectious disease physician with an ophthalmic surgeon, two arguments could be made for the greater duty of the infectious disease physician: this could arise from both their greater skill in managing patients with COVID-19 and by their choice of specialty. It could be argued that by choosing to train in the management of infectious diseases they have implicitly agreed to accept a predetermined level of risk,^4^ and therefore, frontline pandemic work may fall within the scope of agreed duties. In short, the obligation to participate in frontline work is higher for those who chose to ‘opt in’ to higher risk work at specialty training, than for those who chose to ‘opt out’. This neither implies the infectious disease doctor has an absolute duty to participate in frontline work regardless of personal risk or that the ophthalmic surgeon has no duty, rather that the degree of obligation may vary between specialties within certain constraints.

Licenced doctors may not be the only doctors asked to help care for patients during the pandemic. In the UK, the government called for recent retirees and senior medical students to volunteer in the response to COVID-19.^20^ This leads to the question of when professional or vocational obligations start and end. As medical students’ training is subsidised by the UK government, this could be grounds for the start of a duty to society, with this only being able to be realised later in medical school when students may have skills that could aid in the response. Although the age of most medical students means they are likely to be low risk for complications of COVID-19, it is not clear that the skills medical students have are sufficiently useful to counter the perhaps greater risks of psychological and emotional distress in those who have not developed resilience by working in the health system. The duty to return for retirees, or those that have chosen to leave medicine, should not be grounded in their choice to be a doctor. It would be an unduly extensive duty if understood as a lifelong commitment lasting beyond a professional career. However, as recent retirees in acute care specialties could be extremely skilled staff, this duty could be ground in a ‘duty of easy rescue’. This means that ‘if it is in your power to save a life or prevent something bad from happening where the cost to you is negligible, very less, or has comparable moral importance, you are morally obliged to do it’.^21^ However, in the case of COVID-19 retirees are by their age at risk of death and serious illness, challenging the idea that the cost isnegligible or this an ‘easy rescue’. Furthermore, intensive care unit beds and ventilators (as well as doctors) are a finite resource. Putting retirees on the front line may generate a net harm, rather than a net benefit.

## What are the reciprocal obligations to doctors from their employers and patients?

Much of the literature focuses on the duties of doctors and much less is said of what is owed to them in return. Studies have found that doctors feel they have a duty to work only if certain obligations are fulfilled by the state or institution.^5^ This includes basics, such as employer obligations to put measures in place to protect doctors and their families, such as the provision of personal protective equipment (PPE) and of vaccination for themselves or family members (if available).^5^

Evidence also suggests that willingness may not necessarily be increased by the implementation of practical or pragmatic solutions but may be instead more deeply rooted in a number of factors, such as the extent to which doctors feel included in preparedness planning, or various sociodemographic and family issues. These are likely to influence doctors’ willingness to work during a pandemic or other emergency.^5^ Standards of care may have to be adjusted, and the legal repercussions of these adjusted standards need to be addressed.^1^ This includes providing adequate indemnity cover for anyone asked to act outside of their established role.

Lastly, whereas much has been written on what makes a good doctor, less attention has been said about the good patient.^4^ Obligations towards the professional have been suggested to include informing the professional about any known risk of infection,^22^ truthfulness, compliance, tolerance and trust^11^ and to ‘relate to physicians in all of the virtuous ways that govern human interrelationships and social conduct’.^23^ In this pandemic, it is the behaviour of the *potential*, rather than the *actual* patient that is of upmost importance. An existing patient–doctor relationship cannot be the basis of these obligations, because key behaviours for the public include those to prevent them becoming a patient by engaging with infection control measures such as wearing a face covering and social distancing.

## What should doctors do if these reciprocal obligations are not met?

As these reciprocal obligations towards doctors remain implicit and somewhat undefined, this can leave doctors in a difficult position on how to act if they perceive obligations are not met. A clear avenue for doctors to turn to might be their professional bodies, but so far, UK professional guidelines remain remarkably ambiguous as to the expectations of doctors. The apparent failure of employers and the state to meet obligations to doctors has come to the forefront in the UK over shortages and perceived inadequacy of PPE. Doctors have been questioning whether they can refuse to treat patients if they do not have adequate PPE. Here, the General Medical Council’s(GMC) Good Medical Practice advises that ‘Doctors must not refuse to treat patients because their medical condition may put the doctor at risk’, but that all available steps should be taken to minimise that risk before providing treatment, which includes escalating concerns to employers.^24^ Unfortunately, this both places the burden of the moral decision making squarely on the doctor, rather than the employer, and presents a structural problem for doctors who may all too easily be pressured into unacceptable working conditions by employers.

So what should doctors do if finding themselves in such as position? After establishing the obligation is unmet, doctors should be justified in opting out of patient care tasks. However, rather than considering this opting out of a COVID-19 patient care role, this should be considered a task-specific opt out proportionate to the obligation not met. For example, if an emergency physician has access to a fluid-resistant surgical mask, but not to an FFP3 respirator mask, it would be proportionate for that doctor to refuse to do specific high-risk procedures that the mask is necessary for, such as intubation, but not proportionate to refuse to provide any care to a patient at all.^25^ Importantly, this opt out is not specific to caring for patients with COVID-19 but would apply to all healthcare provision tasks that are affected by the COVID-19 pandemic. This could include circumstances such as PPE shortages causing lack of gowns for surgeons. A surgeon would then be justified if they refused to operate if the lack of gown left them at greater risk of contracting a blood-borne virus.

## Conclusion

We have argued that doctors have a duty to participate in pandemic response due to their special skills, but these skills vary between different doctors, and their duties are constrained by other competing rights. In special circumstances such as a pandemic, these obligations may be considered supererogatory (in ethics, an act is supererogatory if it is good but not morally required to be done). This means an opt out policy, based on an assessment of these competing duties, while not desirable would be ethically justifiable.

From both an ethical and pragmatic perspective, doctors must be viewed in the context of rich lives with multiple competing demands. We should encourage doctors to meet the demand for medical aid in the pandemic, but those who make the sacrifices and increased efforts are owed reciprocal obligations in return. When reciprocal obligations are not met, doctors are further justified in opting out of specific tasks, as long as this is proportionate to the unmet obligation.

To encourage doctors to meet the demand for healthcare provision and to prevent structural injustices undermining reciprocal obligations owed to doctors, it is important to explicitly define the reciprocal obligations owed to doctors. We propose the minimum obligations in table 1. Further work is required to define these professional standards that should take into account the capacity for structural factors that may influence doctor’s agency and should aim to meet these reciprocal obligations.

**Table 1:** Key reciprocal obligations owed to doctors

1.	Adequate indemnity insurance and licencing arrangements to be provided by medical bodies; *this is not to prevent doctors acting badly without impunity, but to ensure that the extraordinary contextual matters are taken into account.*
2.	Personal protective equipment, training and clinical supervision to be provided by employers – *Training and supervision may be particularly needed for those acting outside their normal role*.
3.	Sustainable working hours and adequate rest to be mandated by governing bodies and enforced by employers.
4.	Priority testing for those who develop symptoms – *The highest priority testing should be for inpatients or those in high-risk settings (eg, elderly care homes) in which the test result changes clinical outcome, but doctors should be prioritised above other community testing*.
5.	Access to best available medical care if they get sick *– This could include priority (but not guaranteed) access to intensive care units for suspected occupationally acquired COVID-19*.
6.	Sufficient sick pay for occupationally acquired illness or burnout – *Not limited to COVID-19 infection but to other causes of ill health caused by the wider environment*.
7.	Consideration of wider social factors *– This includes employer obligations to provide childcare, other caring responsibilities and transport to work if affected by public transport closures or increased hours*.
8.	Acknowledgement of contribution and service by employers, governments, media and the public – *This includes for extra hours worked and avoiding making doctors scapegoats for unavoidable bad consequences of the pandemic*.
9.	Postpandemic mental health support and leave to be provided by employers – *Workers risk suffering long-term effects and burnout and likely will have not been allowed annual leave during the pandemic. Postpandemic annual leave may need to be prioritised over returning elective services to normal*.
